# Exercise in Pregnancy and Risk of Postpartum Depression: A Randomised Controlled Trial

**DOI:** 10.1111/1471-0528.70010

**Published:** 2025-09-21

**Authors:** Gabriele Saccone, Giorgia Buonomo, Alessandra Ammendola, Luca Bardi, Mariarosaria Motta, Elisabetta Gragnano, Mariavittoria Locci

**Affiliations:** ^1^ Department of Neuroscience, Reproductive Sciences and Dentistry, School of Medicine University of Naples Federico II Naples Italy

**Keywords:** antenatal exercise, maternal mental health, postpartum depression, pregnancy, randomised controlled trial

## Abstract

**Objective:**

To evaluate whether regular aerobic exercise during pregnancy reduces the incidence of postpartum depression in women with low‐risk singleton pregnancies.

**Design:**

Single‐centre randomised controlled trial.

**Setting:**

Department of Obstetrics and Gynaecology, University of Naples Federico II, Italy.

**Population or Sample:**

A total of 398 women with low‐risk singleton pregnancies enrolled during the first trimester of pregnancy.

**Methods:**

Participants were randomly allocated in a 1:1 ratio to an exercise group or control group. The intervention consisted of a structured aerobic exercise programme (three 60‐min sessions per week) from randomisation until 35 weeks' gestation, or earlier if delivery or obstetric complications occurred. The primary outcome was Edinburgh Postnatal Depression Scale (EPDS) score ≥ 12 3 months postpartum. Secondary outcomes included EPDS ≥ 9, clinical diagnosis of postpartum depression (DSM‐V), and maternal/perinatal outcomes. Analyses were performed on an intention‐to‐treat basis, with relative risk (RR) and 95% confidence interval (CI) calculated.

**Main Outcome Measures:**

Incidence of postpartum depression, defined as EPDS ≥ 12 at 3 months postpartum.

**Results:**

Of the 398 participants, 199 were randomised to the exercise group and 199 to the control group. Women in the exercise group had a significantly lower incidence of EPDS ≥ 12 and ≥ 9 at 3 months postpartum compared with controls, as well as lower mean EPDS scores. No significant differences in adverse maternal or perinatal outcomes were observed.

**Conclusions:**

Regular antenatal aerobic exercise significantly reduced the risk of postpartum depression, supporting its role as a preventive strategy in low‐risk pregnancies.

**Trial Registration:**

Clinicaltrials.gov identifier: NCT06355375

## 1 Introduction

Postpartum depression (PPD), also called peripartum depression, is common [1], with an estimated prevalence ranging from 5% to 30% [2]. It is one of the most disabling complications of childbearing, and it is often undertreated and underdiagnosed [3].

Diagnosis of PPD is based on the same diagnostic criteria of major depression, including depressed mood, sleep or food disturbance, fatigue, anhedonia, and others [4, 5]. Suicidal ideation is also common [6]. Treatment and recovery time vary, depending on the severity of the disease [7]. PPD is often treated with psychotherapy [8], along with a pharmacologic approach, including antidepressants [9] such as selective serotonin reuptake inhibitors (SSRIs) [10, 11, 12, 13].

A PPD screening helps diagnose the condition so it can be treated early [14]. Different screening tools have been proposed, with different sensitivity and specificity [15]. The Edinburgh Postnatal Depression Scale (EPDS) is one of the most commonly used [16].

Interventions to reduce PPD have mainly been focused on enhancing screening to increase treatment rates [17], with, so far, unknown effectiveness [18]. Recently, data have been published on the effectiveness of exercise in reducing antenatal depression [19], and in reducing maternal depressive symptoms during the perinatal period [20]. Because antenatal depression is an important predictor of postpartum depression, it has been suggested that antenatal physical activity may be a promising approach to preventing postpartum depression [21].

### 1.1 Objective

The hypothesis of this trial was that in singleton pregnancies, regular aerobic exercise during pregnancy would reduce the rate of PPD.

## 2 Methods

### 2.1 Study Design and Participants

This was a single‐centre open‐label parallel group randomised clinical trial (RCT) of women with singleton pregnancies conducted from March 2024 to April 2025, at University of Naples Federico II (Italy). The trial was approved by the local ethics committee. All participants in the trial provided written informed consent.

Eligible participants were women with singleton low‐risk pregnancies aged between 20 and 40 years, with gestational age at randomisation < 14 weeks.

Exclusion criteria were: multiple gestations, high‐risk pregnancies (e.g., diabetes mellitus, chronic hypertension, known foetal malformations at the time of randomisation, severe obesity, heavy smoking), diagnosis of PPD in a prior pregnancy, prior or current psychiatric disorders, IVF pregnancy, prior preterm delivery, and any contraindication for exercise [22], including vaginal bleeding at the time of randomisation, pessary or cerclage in situ at the time of randomisation, severe lung or heart disease. We also excluded women who already perform agonistic sport activity at the time of randomisation.

Women were enrolled at the time of their first trimester screening between 11 and 13 weeks, after major malformations were ruled out.

### 2.2 Randomisation and Masking

Eligible participants were randomly allocated in a 1:1 ratio to either the exercise group (i.e., intervention group) or the no exercise group (i.e., control group). Women were randomised by a web‐based system to receive the intervention or the control.

### 2.3 Intervention

Women in the intervention group were offered a structured aerobic exercise programme. The training programme was a 3 days per week 60‐min session, from randomisation until 35 weeks. The exercise programme was based on The American College of Obstetricians and Gynaecologists (ACOG) standards [22], and included a gradual warm‐up, aerobic resistance, pelvic floor strengthening, coordination and balance exercise, stretching exercise, and relaxation. To be considered adherent to the intervention, women should perform at least 15 weeks of the programme and a minimum of 65% of the planned workout sessions.

Participants in both groups received general nutritional counselling from the health‐care providers, as per the standard of care. Participants randomised to the control group did not receive any structured exercise programme or specific recommendations regarding physical activity. Their activity levels during pregnancy were not formally assessed.

### 2.4 Outcomes

Self‐reported postpartum depressive symptoms were assessed with the EPDS 3 months after delivery. The scale consists of 10 items scored on a 4‐point Likert scale (0–3) addressing common depressive symptoms [16]. A composite score is calculated by taking the sum of all items, ranging from 0 (absence of depressive symptoms) to 30 (highest score). Women who had a score of ≥ 9 received a closer follow‐up [23] and were sent to the psychiatrist for counselling and a structured clinical interview for DSM–V Disorders (SCID) for the diagnosis of PPD [24].

The primary outcome was EPDS ≥ 12, 3 months after delivery. The secondary outcomes were EPDS ≥ 9, 3 months after delivery; diagnosis of PPD based on DSM‐V; and maternal and perinatal outcomes.

### 2.5 Sample Size Calculation

Calculation of the sample size was based on the following considerations: a prevalence of PPD in our cohort of 10%, a prevalence of EPDS ≥ 12 of 20% [24], and a decrease in the primary outcome of 50% based on regular aerobic exercise in pregnancy.

We determined that a sample size of 398 patients would provide a power of 80% to detect a 50% relative reduction in the primary outcome from a baseline risk of 20%, with a 2‐sided type 1 error of 5%.

### 2.6 Statistical Analysis

Data are shown as means, or as numbers (percentages). Univariate comparisons of dichotomous data were performed with the use of the chi‐squared test with continuity correction. Comparisons between groups were performed with the use of the *T*‐test to test group means by assuming equal within‐group variances. The primary analysis was an intention‐to‐treat comparison of the treatment assigned at randomisation. The effect of antenatal exercise during pregnancy on each outcome was quantified as the unadjusted relative risk (RR) or as the mean difference (MD) with a 95% confidence interval (CI).

In addition to the pre‐specified intention‐to‐treat (ITT) analyses, we conducted post hoc exploratory analyses to assess the effect of adherence on postpartum depressive symptoms.

A 2‐sided *p* value < 0.05 was considered significant.

Statistical analysis was performed using the Statistical Package for Social Sciences (SPSS) (IBM Inc).

## 3 Results

### 3.1 Trial Population

**FIGURE 1:**
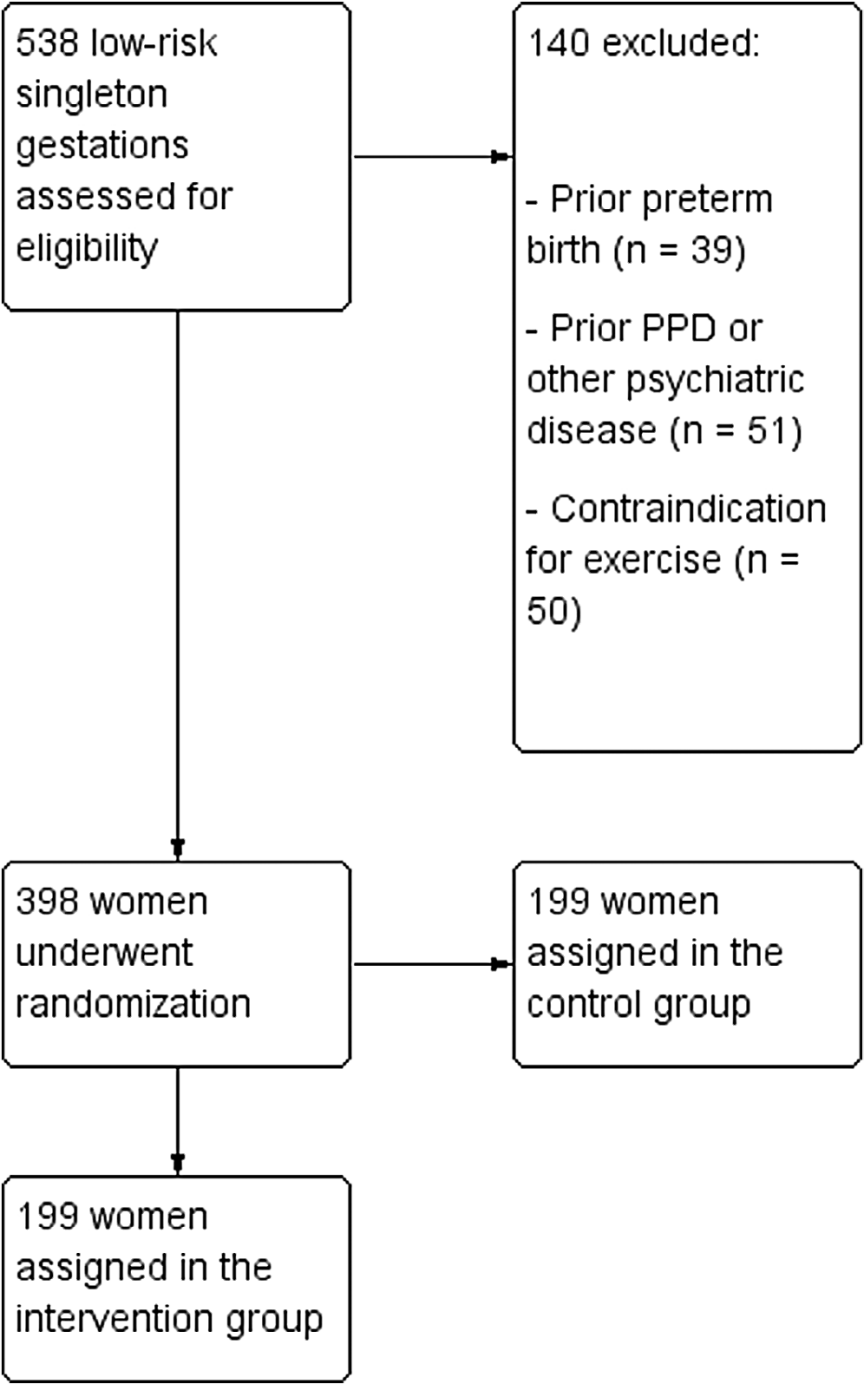
CONSORT study flow‐chart.

During the study period, 398 women agreed to take part in the study, underwent randomisation, and were enrolled and followed up (Figure 1). No patients were lost to follow up for the primary outcome. 199 women (50.0%) were randomised in the exercise group (i.e., intervention group), and 199 women (50.0%) were randomised in the control group. Table 1 shows the baseline demographic and clinical characteristics for each group. All women were singleton gestations and were enrolled in the first trimester of pregnancy at the time of nuchal translucency scan.

**TABLE 1:** Characteristics of the enrolled women.

	Intervention group, *N* = 199	Control group, *N* = 199
Age (years), year	33.9 ± 3.2	33.8 ± 3.1
Prepregnancy BMI	24.0 ± 3.1	23.8 ± 3.3
Planned pregnancy	136 (68.3%)	140 (70.4%)
Nulliparous	108 (54.3%)	100 (50.3%)
Smoking or vape	38 (19.1%)	34 (17.1%)
Education level		
High school diploma or less	48 (24.1%)	53 (26.6%)
University degree	112 (56.3%)	108 (54.3%)
Postgraduate degree	39 (19.6%)	38 (19.1%)
Employment status		
Unemployed	41 (20.6%)	45 (22.6%)
Marital status		
Married or cohabiting	176 (88.4%)	174 (87.4%)
Race		
Caucasian	182 (91.5%)	178 (89.4%)
No. of participants who did not meet adherence criteria^a^	21 (10.6%)	—

*Note:* Data are presented as number (percentage), or as mean ± standard deviation. Sociodemographic variables, including education level, were collected at baseline via self‐report at enrolment.

Abbreviation: BMI, body mass index.

^a^ To be considered adherent to the intervention, women should perform at least 15 weeks of the programme and a minimum of 65% of the planned workout sessions.

21/199 (10.6%) did not meet adherence criteria (Table S1): 12/21 had threatened miscarriage or threatened preterm labour during pregnancy; and 9/21 did not stick with the training programme for personal or no reported reasons.

### 3.2 Maternal and Perinatal Outcomes

Maternal and perinatal outcomes showed no statistically significant differences in GDM, PTB, and rate of caesarean delivery. Women randomised in the exercise group had a higher gestational age at delivery and a significantly lower rate of admission to NICU (Table 2).

**TABLE 2:** Maternal and perinatal outcomes.

	Intervention group, *N* = 199	Control group, *N* = 199	RR or MD (95% CI)
GDM	33 (16.6%)	37 (18.6%)	0.89 (0.58 to 1.37)
Gestational age at delivery, weeks	39.3 ± 1.2	38.9 ± 1.5	**0.40 (0.13 to 0.67)**
PTB < 37 weeks	12 (6.0%)	18 (9.0%)	0.67 (0.33 to 1.35)
Birth weight, g	3233 ± 413	3186 ± 453	32.00 (−53.48 to +117.48)
Admission to NICU	3 (1.5%)	11 (5.5%)	**0.27 (0.08 to 0.96)**
Caesarean delivery	43 (21.6%)	45 (22.6%)	0.96 (0.66 to 1.38)
Exclusive breastfeeding	39 (19.6%)	43 (21.6%)	0.91 (0.62 to 1.33)

*Note:* Data are presented as number (percentage), or as mean ± standard deviation. Bold face data are statistically significant.

Abbreviations: CI, confidence interval; GDM, gestational diabetes mellitus; MD, mean difference; NICU, neonatal intensive care unit; PTB, preterm birth; RR, relative risk.

### 3.3 Depressive Outcomes

Women randomised in the exercise group had a lower rate of EPDS ≥ 12 and ≥ 9 3 months after delivery, and a lower mean EPDS score (Table 3) (Table S2). Post hoc per‐protocol analysis comparing adherent participants with controls is shown in Table S3.

**TABLE 3:** Depressive outcomes.

	Intervention group, *N* = 199	Control group, *N* = 199	RR or MD (95% CI)
EPDS ≥ 12, 3 months after delivery	12 (6.0%)	40 (20.1%)	**0.30 (0.16 to 0.55)**
EPDS ≥ 9, 3 months after delivery	35 (17.6%)	63 (31.7%)	**0.56 (0.39 to 0.80)**
EPDS score, mean	5.1 ± 3.7	7.1 ± 5.2	**−2.00 points (−2.89 to −1.11)**
Postpartum depression^a^	13 (6.5%)	22 (11.1%)	0.59 (0.31 to 1.14)

*Note:* Data are presented as number (percentage), or as mean ± standard deviation. Bold face data are statistically significant.

Abbreviations: CI, confidence interval; EPDS, Edinburgh Postnatal Depression Scale; MD, mean difference; RR, relative risk.

^a^ Postpartum depression diagnosis based on SCID‐5, in women with EPDS ≥ 9.

### 3.4 Trial Adverse Events

No maternal deaths or serious injuries occurred. In the intervention group, minor adverse events included transient musculoskeletal discomfort (reported in 7 participants, 3.5%) and fatigue (5 participants, 2.5%), all of which resolved spontaneously and did not require medical intervention or discontinuation of the programme.

## 4 Discussion

### 4.1 Main Findings

This RCT showed that in low‐risk singleton gestations, regular aerobic exercise during the antenatal period was associated with significant benefits in terms of postpartum depression, with a lower rate of EPDS score ≥ 12. The 10‐item EPDS is the most commonly used depression screening tool in postpartum care, with values of 12 or higher most often used to identify women who might have peripartum depression [16].

Women who performed aerobic exercise in pregnancy had also a lower rate of PPD based on DSM‐V, even if statistical significance was not reached (Table 3). Although the reduction in EPDS scores was statistically significant, the study was not powered to detect differences in the diagnosis of postpartum depression based on DSM‐V criteria, which had a lower incidence. The absence of a significant difference in this outcome should therefore be interpreted with caution and may reflect limited statistical power rather than a true lack of effect.

Our trial also showed that antenatal exercise was associated with better maternal and perinatal outcomes, with a trend for benefits for GDM and preterm birth, and a significantly lower rate of admission to NICU.

This trial has several limitations. The open‐label nature of the trial could have affected medical decision‐making, and therefore the results of the study. Second, the numerous secondary endpoints with no adjustment for multiple comparisons could have led to a type 1 error.

Third, the single centre nature of the trial raises the question of the external generalisability of the findings. Fourth, the incidence of the primary outcome in the control group was lower than expected, with impact on the sample size calculation.

Noncompliance to the intervention protocol was substantial and may have led to underestimations of the possible benefits of exercise. Physical activity in the control group was not monitored or quantified. Therefore, we cannot exclude the possibility that some women in the control group engaged in regular physical activity, potentially attenuating the contrast between groups.

Our study excluded women with a history of psychiatric disorders or conceived through assisted reproductive technologies, such as IVF. While this allowed for a more homogeneous sample of low‐risk pregnancies, it limits the generalisability of our findings to populations that are often at increased risk for postpartum depression. Also, a limitation of our study was the lack of structured assessment for depressive symptoms at baseline. While women with known psychiatric diagnoses were excluded, the presence of subclinical or undiagnosed depression at enrolment cannot be ruled out. We did not explore potential correlations between EPDS scores and parity or obstetric outcomes, as the study was not designed or powered to detect such associations.

The observed incidence of EPDS ≥ 12 in the control group was lower than expected based on prior literature, which may have reduced the statistical power to detect differences in this secondary dichotomised outcome. Despite this, the continuous EPDS score showed a significant reduction in the intervention group, supporting the effect of antenatal exercise on postpartum mood.

### 4.2 Implications

Aerobic exercise improves general health and well‐being, improves cardiorespiratory fitness, and reduces the risk of obesity [25, 26]. In pregnancy, aerobic exercise has been associated with several benefits [22], including a lower incidence of excessive gestational weight gain, gestational diabetes, hypertensive disorders, preterm birth, and caesarean delivery [22, 27, 28, 29, 30, 31]. Recently, compelling evidence showed that physical activity and yoga are associated with benefits in depressed pregnant and postpartum women [32].

Physical activity may reduce depressive symptoms through different biological mechanisms, including increasing beta‐endorphin levels [33], which are associated with improved mood, euphoria, and enthusiasm but also pain reduction [34], and increasing levels of brain neurotransmitters associated with feelings of satisfaction and euphoria [35]. Specific exercise, such as prolonged running, seems also to be associated with an increase in overall dendritic spine density in the brain [36], preparing for future studies on its role in improving memory in patients with Alzheimer's disease [37].

The mechanisms by which antenatal exercise may reduce the risk of postpartum depression are, therefore, likely multifactorial. Exercise promotes neuroplasticity and increases levels of brain‐derived neurotrophic factor (BDNF) [38]. Psychologically, regular activity may enhance self‐efficacy, improve sleep quality, and reduce perceived stress and anxiety [39].

### 4.3 Comparison With Prior Literature

Two prior randomised trials evaluated the effects of antenatal exercise on the risk of postpartum depression [21, 25]. In both studies, the authors were not able to find a significantly lower prevalence of high EPDS scores among women randomised to the antenatal regular exercise programme during pregnancy compared with controls.

In the 2012, Songøygard et al. [25] performed an RCT aimed to evaluate whether exercise during pregnancy reduces the risk of postnatal depression, assessed by EPDS 3 months after delivery. The authors did not find a lower prevalence of high EPDS scores among women randomised to regular exercise during pregnancy compared with the control group. However, a subgroup of women in the intervention group who did not exercise regularly prior to pregnancy had a reduced risk of postnatal depression.

In the 2019, Coll et al. [21] aimed to assess the efficacy of regular exercise during pregnancy on the prevention of postpartum depression. Participants assigned to the intervention were engaged in a 16‐week supervised exercise programme including aerobic and resistance training delivered in 60‐min sessions 3 times per week. They found that moderate‐intensity exercise during pregnancy did not lead to significant reductions in postpartum depression. However, the noncompliance with the intervention protocol was very high in the trial.

Several factors may explain why our trial demonstrated a significant reduction in EPDS score, whereas previous studies have shown more heterogeneous or null results. First, the intervention was initiated in the first trimester, allowing for a longer cumulative exposure to exercise across pregnancy. Second, the exercise programme was structured, supervised, and designed to optimise adherence, which may have enhanced its physiological and psychological benefits. Third, the study population was composed of low‐risk women, possibly resulting in a more consistent response to the intervention. These methodological elements may have contributed to the observed effect.

## 5 Conclusions

In summary, in low‐risk singleton pregnancies, regular aerobic exercise during the antenatal period led to a significant reduction in postpartum depression.

## Author Contributions

All authors contributed equally.

## Disclosure

The authors have nothing to report.

## Ethics Statement

This study was approved by the local IRB of the University of Naples Federico II, number 02/24 (approved on 23.02.24).

## Conflicts of Interest

The authors declare no conflicts of interest.

## Supporting information


**Data S1:** bjo70010‐sup‐0001‐Tables.docx.

## Data Availability

The data that support the findings of this study are available from the corresponding author upon reasonable request.
