# Supplementary material for: Exercise in Pregnancy and Risk of Postpartum Depression: A Randomised Controlled Trial
Source: BJOG. 2025 Sep 21;133(2):211–7. doi: 10.1111/1471-0528.70010 (PMC12678040; doi:10.1111/1471-0528.70010)
Supplement: Supplementary file 1 — Data S1: bjo70010‐sup‐0001‐Tables.docx. [file BJO-133-211-s001.docx]

**SUPPLEMENTARY TABLES**

**Table S1.** Baseline characteristics of the intervention group stratified by adherence

|  | **Non-adherent**  **N = 21** | **Adherent***  **N = 178** |
| --- | --- | --- |
| **Age (years), year** | 34.1±3.9 | 33.9±3.1 |
| **Prepregnancy BMI** | 23.6±3.2 | 24.0±3.1 |
| **Planned pregnancy** | 15 (71.4%) | 121 (68.0%) |
| **Nulliparous** | 11 (52.4%) | 97 (54.5%) |
| **Smoking or vape** | 4 (19.0%) | 34 (19.1) |
| **Education level**   - **High school diploma or less** - **University degree** - **Postgraduate degree** | 8 (38.1%)  10 (47.6%)  3 (14.3%) | 40 (22.5)  102 (57.3)  36 (20.2%) |
| **Employment status**   - **Unemployed** | 4 (19.0%) | 37 (20.8%) |
| **Marital status**   - **Married or cohabiting** | 19 (90.5%)) | 157 (88.2%) |
| **Race**   - **Caucasian** | 19 (90.5%) | 163 (91.6%) |

*Data are presented as number (percentage), or as mean ± standard deviation*

*BMI, body mass index*

*Sociodemographic variables, including education level, were collected at baseline via self-report at enrollment*

*** *To be considered adherent to the intervention, women should perform at least 15 weeks of the program and a minimum of 65% of the planned workout sessions.*

**Table S2.** Distribution of responses to each item of the Edinburgh Postnatal Depression Scale (EPDS) at 3 months postpartum, stratified by randomized group (Exercise vs Control).

| **EPDS Item** | **Response** | **Exercise group**  **N = 199** | **Control Group**  **N = 199** |
| --- | --- | --- | --- |
| **Q1.** I have been able to laugh and see the funny side of things. |  |  |  |
|  | 0 | 127 | 98 |
|  | 1 | 50 | 60 |
|  | 2 | 18 | 30 |
|  | 3 | 4 | 11 |
|  |  |  |  |
| **Q2.** I have looked forward with enjoyment to things. |  |  |  |
|  | 0 | 131 | 96 |
|  | 1 | 43 | 57 |
|  | 2 | 17 | 31 |
|  | 3 | 8 | 15 |
|  |  |  |  |
| **Q3.** I have blamed myself unnecessarily when things went wrong. |  |  |  |
|  | 0 | 109 | 91 |
|  | 1 | 56 | 57 |
|  | 2 | 24 | 35 |
|  | 3 | 10 | 16 |
|  |  |  |  |
| **Q4.** I have been anxious or worried for no good reason. |  |  |  |
|  | 0 | 98 | 72 |
|  | 1 | 54 | 56 |
|  | 2 | 30 | 42 |
|  | 3 | 17 | 29 |
|  |  |  |  |
| **Q5.** I have felt scared or panicky for no very good reason. |  |  |  |
|  | 0 | 104 | 77 |
|  | 1 | 54 | 51 |
|  | 2 | 25 | 43 |
|  | 3 | 16 | 28 |
|  |  |  |  |
| **Q6.** Things have been getting on top of me. |  |  |  |
|  | 0 | 111 | 86 |
|  | 1 | 47 | 50 |
|  | 2 | 28 | 40 |
|  | 3 | 13 | 23 |
|  |  |  |  |
| **Q7.** I have been so unhappy that I have had difficulty sleeping. |  |  |  |
|  | 0 | 120 | 87 |
|  | 1 | 45 | 54 |
|  | 2 | 24 | 40 |
|  | 3 | 10 | 18 |
|  |  |  |  |
| **Q8.** I have felt sad or miserable. |  |  |  |
|  | 0 | 117 | 85 |
|  | 1 | 48 | 55 |
|  | 2 | 25 | 41 |
|  | 3 | 9 | 18 |
|  |  |  |  |
| **Q9.** I have been so unhappy that I have been crying. |  |  |  |
|  | 0 | 123 | 89 |
|  | 1 | 42 | 53 |
|  | 2 | 25 | 41 |
|  | 3 | 9 | 16 |
|  |  |  |  |
| **Q10.** The thought of harming myself has occurred to me. |  |  |  |
|  | 0 | 114 | 105 |
|  | 1 | 53 | 52 |
|  | 2 | 23 | 27 |
|  | 3 | 9 | 15 |

**Table S3.** Depressive outcomes. Post-hoc per-protocol analysis comparing adherent participants in the intervention group with the control group

|  | **Non-adherent**  **N = 21** | **Adherent***  **N = 178** | **RR or MD (95% CI)** |
| --- | --- | --- | --- |
| **EPDS > 12, three months after delivery** | 0 | 12 (6.7%) | 0.33 (0.02 to 5.31) |
| **EPDS > 9, three months after delivery** | 2 (9.5%) | 33 (18.5%) | 0.51 (0.13 to 1.99) |
| **EPDS score, mean** | 4.6±3.3 | 5.1±3.7 | -0.50 points (-2.01 to 1.01) |
| **Postpartum depression**** | 1 (4.8%) | 12 (6.7%) | 0.71 (0.10 to 5.16) |

*Data are presented as number (percentage), or as mean ± standard deviation*

*EPDS, Edinburgh Postnatal Depression Scale*

*RR, relative risk; MD, mean difference; CI, confidence interval*

*** *To be considered adherent to the intervention, women should perform at least 15 weeks of the program and a minimum of 65% of the planned workout sessions.*

***Postpartum depression diagnosis based on SCID-5, in women with EPDS > 9*
